# Possession Syndrome in Rural Nepal: A Case Study Examining Cultural, Clinical and Forensic Implications

**DOI:** 10.1155/crps/6680684

**Published:** 2025-12-12

**Authors:** Alok Atreya, Sabbu Maharjan, Samata Nepal, Ajay Risal, Sneha Chaudhary, Namuna Rasaely

**Affiliations:** ^1^ Department of Forensic Medicine, Lumbini Medical College, Palpa, Nepal, lmc.edu.np; ^2^ Department of Psychiatry, Lumbini Medical College, Palpa, Nepal, lmc.edu.np; ^3^ Department of Community Medicine, Lumbini Medical College, Palpa, Nepal, lmc.edu.np; ^4^ Department of Psychiatry, Kathmandu University School of Medical Sciences, Dhulikhel, Nepal, kusms.edu.np; ^5^ Lumbini Medical College, Palpa, 32500, Nepal, lmc.edu.np

**Keywords:** cultural psychiatry, dissociative disorders, forensic psychiatry, mental health, Nepal, possession syndrome

## Abstract

Possession state is a disorder of consciousness with substitution of the personality, which is claimed to be a spirit, a deity, a dead person or some other power. In rural Nepal these experiences are normalised and Hindu communities often attribute psychological conditions to a supernatural cause. We report the case of a 30‐year‐old woman who presented with acute‐onset symptoms characterised by episodes of altered consciousness, vocalisations suggestive of religious chants, and deity‐associated behaviour, probably influenced by local suggestions of divine possession. Additional notable features included similar presentations among a family member and seeking help from traditional healers prior to psychiatric consultation. Medical examinations and investigations were normal. Specific cultural and religious manifestations posed challenges to clinical interpretation. The patient responded well to combined pharmacotherapy and supportive psychotherapy during her brief hospital stay, with cessation of possession episodes. This case report highlights the importance of cultural competence in Nepalese forensic psychiatric evaluations, particularly in the context of possession states, while examining the application of mental health legislation in traditional cultural settings.

## 1. Introduction

Trance and possession states remain enigmatic historical phenomena that integrate spiritual, social, and psychological perspectives. Trance is a temporary disorder of consciousness without personality substitution; possession, by contrast, is the disorder of consciousness with the substitution of the personality, which is claimed to be a ‘spirit/deity/dead person/other power’. In rural Nepal, these experiences are normalised; in particular, Hindu communities attribute psychological conditions to a supernatural cause, and thus these experiences remain intertwined with the cultural fabric. It is classified under dissociative disorders in Diagnostic and Statistical Manual of Mental Disorders (DSM‐5) and as a possession and trance disorder in the International Classification of Disease (ICD‐11) [1]. Despite these classifications, diagnostic challenges persist in discerning culturally normative behaviours from pathological conditions, particularly in forensic contexts. This case report aims to elucidate this complexity by examining its socio‐cultural significance, psychological basis and implications for clinical practice.

## 2. Case Presentation

A 30‐year‐old married woman, mother of one child, educated up to secondary school, belonging to a Hindu nuclear family, from a middle socio‐economic background residing in the mountainous region of Nepal and with a husband that worked abroad, was brought to an out‐patient department (OPD) of psychiatry by her sister with complaints of episodes of altered consciousness with abnormal behaviour for the past 7 days. The sister said the episodes began following news of a nearby female resident possessed by ‘Lord Shiva’, a Hindu deity.

At first, the patient experienced tingling sensations all over her body; then, her body began to shake uncontrollably. She started chanting ‘om namah shivaye*’* repeatedly, and shouting that ‘Lord Shiva’ was in her, and that she would destroy anyone who harmed her. The episode lasted approximately half an hour, with the patient reporting only partial recall of events.

Post‐episode, the patient showed marked behavioural changes, including adopting a strict vegetarian diet and an increased frequency of visiting temples. Still her mood remained stable, sleep was adequate and she performed daily activities as before. However, the patient had other similar multiple episodes that usually occurred on mentioning of God and visiting temples. There were also unprovoked episodes, after which patient was taken to a faith healer without any improvement and then was brought to our OPD for management. During evaluation in OPD, patient experienced another episode where she said there were multiple idols of God and acted strangely by protruding her tongue and again asserting that she was God. This episode lasted a few minutes and terminated on its own without any intervention.

The patient and her sister reported significant distress associated with these episodes. The patient described feelings of anxiety and bewilderment concerning the involuntary nature of the experience, particularly given her limited recollection of the details. The sister reported family concerns about the patient’s wellbeing and the erratic presentation of symptoms and episodes. The patient maintained her basic daily functioning between episodes. However, the family noted social embarrassment and a reluctance to attend social gatherings due to fear of episode recurrence in public settings.

### 2.1. Medical and Psychiatric History

The patient had no significant past medical or psychiatric history. However, there was a history of similar episodes of possession by deities, but she had not received any treatment.

### 2.2. Clinical Examination and Investigations

To exclude any other medical illness, substance use or neurological causes that could have explained her symptoms, a detailed physical and neurological examination were performed with unremarkable findings. Laboratory investigations, including complete blood count, liver function tests, and kidney function tests, were normal. Electroencephalography (EEG) and computed tomography (CT) scans of the brain were conducted to rule out neurological conditions, with normal results.

In the non‐possession state, the mental status examination showed that cognitive functions were well preserved, with no apparent impairment in mood, perception, memory, orientation or judgment.

During the psychiatric evaluation, a formal risk assessment was performed. The patient denied suicidal ideation, intent or plan in her non‐possession state. During possession episodes, no acts of self‐harm or aggression against others were reported. Although she exhibited agitation and made proclamations of ‘destroying’ those who harmed her, these were verbal threats without any physical aggression. Her tongue protrusion and unusual posturing posed no safety risks. Her sister confirmed no history of violence during current or previous episodes. The patient was assessed as having a low risk of self‐harm or harming others; however, close monitoring during episodes was advised to avert unintentional injury.

### 2.3. Treatment and Follow‐up

The patient received inpatient psychiatry care for a period of 7 days. Concurrently, oral treatment was initiated with escitalopram 10 mg daily and clonazepam 0.25 mg daily, to alleviate the symptoms of anxiety and insomnia. The patient received supportive psychotherapy with psycho‐educational components during her hospital stay. The therapeutic approach included psychoeducation on dissociation and stress response, analysis of the patient’s domestic strain while her husband worked abroad, coping strategies to help her recognise and manage early warning signs of dissociative episodes, and cognitive restructuring to integrate cultural and psychological perspectives of her experiences. During the admission, the patient showed marked improvement. She engaged in therapy sessions. She exhibited a clear grasp of the stress‐dissociation connection and felt increasingly self‐assured and self‐controlled. The therapist noted that the patient’s episodes ceased during hospitalisation, which may have been related to the removal of environmental triggers, pharmacological intervention, or therapeutic support. However, due to early discharge and subsequent loss of follow‐up, long‐term therapeutic outcomes could not be evaluated. The proposed course of action was to continue weekly individual psychotherapy sessions focused on stress management, dynamics of marital relationships and cultivating adaptive coping mechanisms, but this could not be implemented.

## 3. Discussion

In the present case, the patient presented with a sudden onset of symptoms, including auditory and visual hallucinations, trance‐like states, and possession related behaviours. These manifestations can be understood through the DSM‐5 criteria, although contextualised within the cultural milieu. Focusing on this dual lens is critical in ensuring that misdiagnosis does not occur, and in providing culturally sensitive treatment practices.

Multiple factors including cultural, family and individual psychological factors were at play throughout the period under observation in this patient. Her clinical picture was a product of the local cultural landscape and religious background, particularly the local understandings and explanations of being divinely possessed. The particularity of her symptoms, including dietary restrictions, compulsive behaviours and specific causal factors, clarified the complex character of her condition. The culturally sanctioned possession experience was also evaluated with family members to differentiate it from other psychiatric disorders, which may require medical attention.

A previous Nepalese study suggested that dissociative disorders are mainly characterised by trance and possession, conditions that are quite common in adult women aged between 20 and 30 years [2], as it was in our case. This age group also includes youths experiencing role transitions and associated psychosocial stressors characteristic of marriageable age in Nepal. The manifestation of this disorder is strongly influenced by cultural and religious beliefs, which may interact with gender‐specific social roles and restrictions. Various frameworks have been proposed to explain this disorder, including stress arising from social and psychological factors, cultural beliefs and learned behaviours that are accepted within specific communities, difficulties in expressing emotional challenges, and the more recent impact of migration‐related cultural adjustments [1, 3]. From a psychiatric perspective, possession states may represent a dissociative defence mechanism where unconscious psychological responses to fear, trauma, and stress manifest through culturally‐influenced expressions and behaviours [4]. In the present case, the patient was managing the house and child alone due to her husband working abroad leading to stress from single parenting and maintaining a long‐distance marriage. Interestingly, another woman living nearby had experienced similar syndrome before the onset of symptoms in our patient after which she had the episode which then continued for weeks. This emphasises the potential role of social contagion and shared cultural and environmental influences in the disorder [5]. Furthermore, even the patient’s sister had a documented history of episode of possession a few years earlier. Familial possession syndrome has been reported in a case report from India where a father exhibiting possession syndrome whose children later developed similar symptoms, suggesting the influence of familial dynamics [4]. This show the significance of recognising the role of family structures and cultural contexts in the emergence and perpetuation of dissociative and possession disorders.

Weintraub and Bui [6] presented a case of a 53‐year‐old man who had experienced recurrent episodes of demonic possession, with symptoms including a demonic voice, superhuman strength and non‐suicidal self‐injury. The case highlighted the significance of cultural context in assessing dissociative presentations with spiritual elements [6]. It was recommended to incorporate a spiritually focused approach within a multidisciplinary treatment plan, particularly when conventional methods prove ineffective [6].

Khan and Sahni [7] reported a case of possession syndrome at high altitude (4575 m/15,000 ft) in a 20‐year‐old Hindu woman, with abnormal behaviour, unrelenting symptoms and limited benefit from usual treatment. Exorcism offered symptomatic relief, and the patient resumed normal activities with no recurrence in a 12‐month follow‐up [7]. This case sheds light on the importance of considering environmental factors, such as high altitude, in the manifestation of possession syndrome [7]. The authors emphasise the need for a holistic approach that integrates medical and theological perspectives to complement existing concepts [7].

Gadit [8] presented a case of a 21‐year‐old woman who presented with episodes of extraordinary strength, aggression, speaking in a foreign language, and bizarre behaviour, with amnesia for these events and no remission despite psychotropic medications.

In Nepal, many patients initially consult traditional faith healers or shamans, resulting in delayed presentation to medical facilities and prolonged intervals, often exceeding 2 weeks before receiving conventional medical intervention [9, 10]. Consistent with these findings, our patient also had delayed seeking medical attention after seeing a faith healer. These observations point to the need for community education programs and collaborations with faith healers to bridge gaps in understanding and facilitate timely interventions [9].

The National Criminal Procedure (Code) Act, 2017 of Nepal is a legal platform that addresses mental illness in the context of criminal responsibility [11]. The Act offers the defence of statutory immunity to a person who, by reason of mental illness at the time of the conduct, could not have the perception of his or her actions and could not decide what was right or wrong [11]. The provision is most practically applicable when possession syndrome has been used as a legal defence. It permits consideration of a more sophisticated assessment as it pertains not just to what the defendant knew, but also how cultural influences may colour that knowledge. The Act gives considerable weight to an independent expert psychiatric evaluation to determine if a defendant had a sound mind when committing a crime [11]. This step is to make certain that individuals with genuine mental health problems receive treatment rather than imprisonment. This provision is in accordance with international standards regarding civil rights exercised by people with mental illness.

Scholars examining possession syndromes have established key dimensions that provide framework for these manifestations. Cultural sensitivity is important to start with, as possession syndrome is so culturally bound that culturally aware knowledge and practices are necessary for its accurate diagnosis and effective treatment. As family predisposition and family dynamics also influence the syndrome in some families, awareness of these factors is crucial in conducting comprehensive evaluations and subsequent treatment. The psychiatric manifestations of possession syndrome can be wide‐ranging, but generally include hallucinations, trance‐like and dissociative states, and abnormal behaviour, with differential diagnosis based primarily on careful differentiation between culturally normative and pathological behaviour. Nepal’s legal system has an umbrella within which such mental matters were then incorporated into the country’s judicial framework by making mental assessments part of a process determining mental competence for defence protection. Ultimately, excellent management necessitates a multidisciplinary approach that addresses the medical, psychological and cultural aspects of the condition collectively. Synergistic cooperation with faith healers and community awareness programmes will create perceptions that promote the replacement of gaps in treatment communications and timely treatment.

This case report contributes to the limited literature on possession syndrome in several ways. First, the case shows a temporal association between the episode of possession of a neighbour and the onset of the patient’s symptoms, demonstrating the likelihood of social contagion in dissociative disorders within close‐knit communities. Second, the possession episode of the patient’s sister augments the existing understanding of familial aggregation, suggesting both psychological modelling and shared genetic vulnerability as possible causes of dissociative responses. The report also presents a pragmatic approach to providing culturally responsive psychiatric services, addressing the clinical challenge of distinguishing mental illness from accepted cultural practices and emphasises the need for collaborative models that integrate traditional healers into mental health referral pathways in rural Nepal in order to accelerate resolution of symptoms where traditional healing had failed.

This case report has some limitations. The patient was lost to follow up after discharge, which limited our ability to assess long‐term treatment outcomes and symptom trajectory. Without the follow up, we could not evaluate whether the resolution of symptoms was maintained or possession episodes recurred. The brief hospital stay also limited our ability to conduct a comprehensive psychotherapeutic intervention in rural setting. As a single case report from one cultural context, generalizability may be limited. Despite these limitations, the present case offers valuable insights into the clinical presentation and treatment of possession syndrome in rural Nepal.

## 4. Conclusions

The case presents how cultural beliefs interact with psychiatric presentation in rural Nepal through social contagion and family patterns in possession syndrome. It advocates the need for an integrated approach to traditional healing and modern psychiatric care, as this presentation was after that which rendered delayed definitive help. This case illustrates that while possession syndrome is a difficult condition to manage, understanding both cultural context and clinical presentation is crucial, particularly in Nepal, where mental health and legal frameworks are in their infancy.

## Ethics Statement

Institutional policy waives ethics committee approval for case reports.

## Consent

Written informed consent was obtained from the patient in her non‐possession state and after she was found to be compos mentis for the publication of the case report.

## Conflicts of Interest

The authors declare no conflicts of interest.

## Funding

This research received no specific grant from any funding agency in the public, commercial, or not‐for‐profit sectors.

## Data Availability

Data sharing not applicable to this article as no datasets were generated or analysed during the current study.
